# Turnover Intention among Field Epidemiologists in South Korea

**DOI:** 10.3390/ijerph17030949

**Published:** 2020-02-04

**Authors:** Sukhyun Ryu

**Affiliations:** Department of Preventive Medicine, College of Medicine, Konyang University, Daejeon 35365, Korea; gentryu@onehealth.or.kr

**Keywords:** occupational stress, satisfaction, turnover, job stress, workforce

## Abstract

The purpose of this study was to explore the level of occupational stress, job satisfaction, and turnover intention among Korean field epidemiologists, and to identify the factors that contribute to their turnover intention. We surveyed the Korean field epidemiologists in the cohort from 2016 to 2018 using the Occupational Stress Inventory, revised edition, and questionnaires developed from the Public Health Workforce Interest and Needs Survey. Fisher’s exact test was used to identify the association between sociodemographic characteristics, occupational stress, job satisfaction, and turnover intention. Overall, 17 Korean field epidemiologists participated in this study (response rate: 74%). More than half of field epidemiologists had turnover intention (53%), and it was less likely to be present in the field epidemiologists recruited from the civilian sector than those recruited from the military (adjusted odds ratio, 0.59; 95% confidence interval, 0.39–0.88). Furthermore, about two-thirds of field epidemiologists had a burden of occupational stress on Role Ambiguity (65%), and only one respondent expressed satisfaction with the job. There was no significant relation among the levels of occupational stress, job satisfaction, and turnover intention. In this study, the field epidemiologists recruited from the military were more likely to have turnover intention. Additional studies to identify possible ways to reduce turnover intention among the public health workforce are warranted.

## 1. Introduction

A field epidemiologist is a professional who applies the various epidemiologic tools to investigate the unexpected spread of infectious diseases in a time-constrained manner [1]. Many countries have initiated Field Epidemiology Training Programmes (FETPs) [2]. In 1999, the Korean FETP began to train the first group of military doctors for the position of field epidemiologist in the two-year full-time curriculum [3,4]. In October 2015, after the outbreak of Middle East Respiratory Syndrome in Korea, the FETP was opened to the public. Since 2016, field epidemiologists have been recruited among civilians as well as military doctors for the temporary two-year term position. 

The primary duties of the field epidemiologist in Korea include investigation of outbreaks, design and operation of the surveillance program in the community, and the conducting of field research to identify risk factors of communicable diseases [5,6,7,8,9,10]. In the case of a communicable disease outbreak, field investigation often begins without a clear hypothesis and requires immediate response to identify the effective measure to control the complicated situation, which requires good communication skills [11]. The role of Korean field epidemiologists in the provincial or metropolitan government, whose affiliations are separated from the Korean Centers for Disease Control and Prevention (KCDC), can be very demanding because they often endure mandatory 24/7 on-call duties, professional isolation from the managerial staff of the department, and limited supervision and management support.

The average turnover rate of Korean field epidemiologists was 40% (45% for civilian, 37% for military) in the 2016–2017 cohort. The high turnover rate causes a considerable managerial cost, including replacement, recruitment, selection, and training of newly employed staff, and loss of organizational knowledge [12,13,14]. Despite the problem of exposure of the field epidemiologists to the very demanding environment and high turnover rate, there is no turnover-related literature available that specifically targets this group of professionals. The current study provides, for the first time, the status of turnover intention and the associative analysis of occupational stress, job satisfaction, and turnover intention among the field epidemiologists in Korea.

## 2. Materials and Methods 

This was a cross-sectional study using the data obtained through a survey of Korean field epidemiologists employed from the provincial or metropolitan government from the years 2016 to 2018. The questionnaires were distributed to all currently active field epidemiologists on October 1, 2018. An anonymous self-administered questionnaire was used to assess the level of occupational stress, job satisfaction, and turnover intention. This study was approved by the ethics committee of the Institutional Review Board designated by the Korean Ministry of Health and Welfare (P01-201809-22-006). Written informed consent was obtained from all study participants.

### 2.1. Demographics

The demographic variables that were collected included age, sex, type of recruitment (physician from military or civilian sectors), length of employment, level of education, marital status, behavior customs such as smoking and drinking, and level of job demand including the number of calls from the field per day and the number of field deployments per week.

### 2.2. Occupational Stress

Occupational stress was measured using the Occupational Stress Inventory, revised edition (OSI-R), which has been well validated in other studies [15,16] and represents a measure of three domains of occupational adjustment (occupational stress, psychological strain, and coping resources). Occupational stress was assessed by the Occupational Roles Questionnaire (ORQ) scale, which is a subscale of the OSI-R [17]. The ORQ consists of questions arranged in the six sections including Role Overload, Role Insufficiency, Role Ambiguity, Role Boundary, Responsibility, and Physical Environment, with ten items per section. The higher the score of ORQ, the stronger the level of stress is (Table A1 in Appendix A). In this study, the internal consistency (Cronbach’s alpha) for the occupational stress was 0.77 (Role Overload), 0.78 (Role Insufficiency), 0.78 (Role Ambiguity), 0.77 (Role Boundary), 0.78 (Responsibility), and 0.77 (Physical Environment). This indicated that the subscales had good internal consistency [18].

### 2.3. Job Satisfaction and Turnover Intention

Self-perceived job satisfaction was examined by a single item, “How do you feel about your job?” and assessed by a 5-point scale, between 1 (extremely dissatisfied) and 5 (extremely satisfied) [19]. The previous studies demonstrated good reliability and validity of a single item-measure of job satisfaction [20,21].

The turnover intention was assessed by providing two optional statements “Planning to or leaving before the assignment period for another job” and “Not planning to leave before the assignment,” which is a modified version of the questionnaire used in the U.S. public health workforce interest and needs survey 2017 [22].

### 2.4. Statistical Analysis

We used descriptive statistics to demonstrate the sociodemographic, turnover intention, level of occupational stress, and job satisfaction of the study population. Fisher’s exact test was used to identify the association between proportions of sociodemographic characteristics, occupational stress, and job satisfaction with turnover intention. To further identify factors associated with turnover intention, logistic regression analysis was performed. In this study, the statistical power for the Fisher’s exact test and logistic regression analysis was 7%. All statistical analysis was performed using R version 3.2.4 (R Foundation for Statistical Computing, Vienna, Austria). All statistical tests were two-sided, and a *p*-value < 0.05 was considered as statistically significant.

## 3. Results

Among 23 Korean field epidemiologists, 17 voluntarily enrolled in this study, corresponding to a response rate of 74%. The average age of study participants was 36 years (range of min–max: 26–58 years). By the type of recruitment, ten (59% of total participants) were recruited from the military and seven were from the civilian sector. By the duration of the employment, seven (41%) were employed less than a year, six (35%) were employed between one and two years, and four (23.5%) had been employed for two years or more. By the education level, twelve (71%) had a bachelor’s degree, three (18%) held a master’s degree, and two (12%) had a doctorate. Eight (47%) were married, and 16 (94%) were non-smoker. Twelve (71%) had a social habit of drinking alcohol. By the number of calls, ten (59%) had less than three calls per day, four (24%) were called four to five times per day, and three (18%) had more than six calls per day. By the number of field deployments for the investigation of a possible outbreak, five (29%) were deployed once a week or less, twelve (71%) had two to three deployments per week. The only parameter that showed a statistical significance of association with turnover intention was a type of recruitment (*p* = 0.02). More than half of the field epidemiologists (53%) expressed an intention, with strong prevalence among the epidemiologists recruited from the military sector (odds ratio, 0.05; 95% confidence interval, 0.01–0.19) (Table 1). No significant associations were identified between turnover intention and other sociodemographic aspects or the level of job demand, including the number of calls per day and the number of field deployments per week.

**Figure 1:**
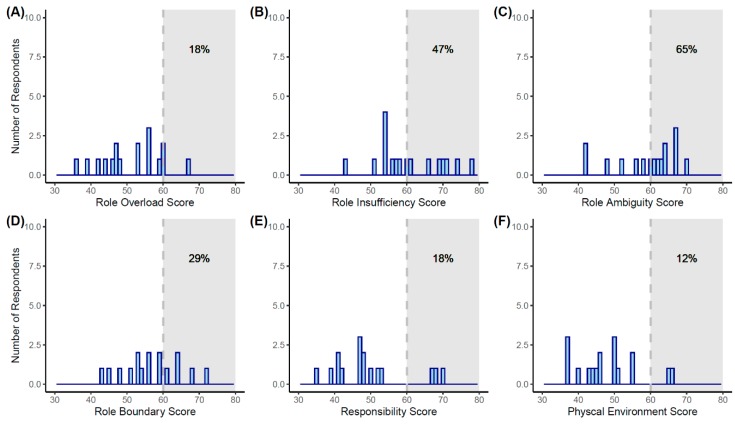
Occupational stress of each subdomain among the study population. Histograms depicting score frequency by the subdomains of (**A**) Role Overload, (**B**) Role Insufficiency, (**C**) Role Ambiguity, (**D**) Role Boundary, (**E**) Responsibility, and (**F**) Physical Environment. The shaded areas in which the T-score is larger than 60 indicate the presence of occupational stress in each subdomain.

Based on OSI-R normative data, 47% (Role Insufficiency), 29% (Role Boundary), 18% (Role Overload and Responsibility), and 12% (Physical Environment) of respondents had been experiencing occupational stress. However, more than half (65%) of the study population had a burden of occupational stress on Role Ambiguity (Figure 1).

Among the subdomain of occupational stress, Role Boundary was significantly associated with turnover intention (*p* = 0.049). However, no significant associations were identified between turnover intention and Role Overload, Role Insufficiency, Role Ambiguity, Responsibility, or Physical Environment (Table 2). Only one respondent expressed satisfaction with the job, in contrast to four respondents that reported dissatisfaction with their employment conditions. However, there was no significant association between job satisfaction and turnover intention (Table 2).

The result of ordinal logistic regression analysis to further examine the significantly associated factors is shown in Table 3. The type of recruitment was the only factor that was significantly associated with turnover intention (adjusted odds ratio, 0.59; 95% confidence interval, 0.39–0.88).

## 4. Discussion

In the light of growing demand for providing a quick and competent response to communicable diseases, the evaluation of the turnover intention and identification of relevant associated factors are crucial for retention and continuing operation of the professional workforce [14]. However, the long-term sustainability of training and operation of field epidemiologists is one of the biggest challenges across many countries [23,24]. In an attempt to address this problem, the current study was designed with the aim of identifying the possible contributory factors to the turnover intention in health professionals along with the previously known related factors, such as sex, age, length of employment, level of job demand, occupational stress, and job satisfaction [14,25,26].

A recent study reported the burden of occupational stress on Role Overload and Physical Environment among field epidemiologists in Southeast Asia and Western Pacific Regions was 56% and 53%, respectively [16]. In the current study, there was less burden of occupational stress on Role Overload and Physical Environment. However, more than half of field epidemiologists that participated in the current study had the burden of occupational stress in Role Boundary; it is likely because the Korean field epidemiologists often have two authority lines from KCDC and the affiliated provincial or metropolitan government. Furthermore, more than half of the study participants had expressed the intention for turnover, an alarming fact by itself. Although the study was unable to determine the significant association between occupational stress, job satisfaction, and type of recruitment, a significant association between turnover intention and type of recruitment was identified. A possible explanation of high turnover intention among field epidemiologists from the military sector is that Korean men with a medical degree who usually trained as physicians mandatorily serve in military or government institutions for three years. These individuals are more likely to have the intention to leave the epidemiologist job to pursue clinical practice, and this may strongly affect the turnover intention of field epidemiologists from the military more than from the civilian sector. 

Previous studies demonstrated that level of job demand, occupational stress, and low job satisfaction was associated with high turnover intention [26,27,28,29]. However, in this study, these were not associated significantly with the turnover intention. The unmeasured factors in the study, such as lower level of working hours and appropriate level of the income compared with other health professionals in Korea, may affect the result. 

The Training Programs in Epidemiology and Public Health Intervention Network recommends the country’s training program of the field epidemiologist as well as the human resources be supported and integrated with the Ministry of Health [30]. However, in South Korea, provincial and metropolitan governments directly recruit field epidemiologists, and some local governments have difficulty in recruiting among civilian sectors. Therefore, recruitment from the military sector is still a viable option. A previous study demonstrated that university-affiliated field epidemiologists are associated with reducing certain subdomains of occupational stress [16]; therefore, to recruit from the university-affiliated, not from military-affiliated personnel, may be one of the possible options to run the Korean program of field epidemiologists. However, more studies are required to identify possible ways to reduce turnover intention among Korean field epidemiologists.

The strength of this study is that this is the first study conducted in Korea that assessed the association between occupational stress, job satisfaction, and turnover intention among the public health workforce and explored the level of job demand, occupational stress, and job satisfaction as well as turnover intention. Therefore, this study provides useful information on the developing managerial strategy for countries that have various types of recruitment for the public health workforce including field epidemiologists. 

Nevertheless, the current study has limitations. First, this study has low statistical power due to the small number of the study population. Second, a possible response bias due to the reliance on self-reporting may have influenced the results. Third, study results may have been affected by personal characteristics and personal traits that were not taken into consideration. Although these biases likely influenced the study results, the findings in this study are of importance for the guidance of implementation of larger scale studies in other public health sectors and the globe.

## 5. Conclusions

In the current study, field epidemiologists recruited from the military may be more prone to having turnover intention than those recruited from the civilian sector. Additional studies to develop the optimal strategies for the recruitment of the public health workforce are warranted.

## Figures and Tables

**Table 1:** Demographics of the study population and association with turnover intention.

Variables	Turnover Intention		Total (*n* = 17) % (*n*)	Odds Ratio	95% CI ^†^	*p*-Value
	No (*n* = 8)	Yes (*n* = 9)				
Age, year						
25–29	0	3	3 (17.6)	-	-	
30–39	4	6	10 (58.8)	-	-	
More than 40	4	0	4 (23.5)	-	-	0.21
Sex						
Male	6	8	14 (82.4)	1 (Reference)	-	
Female	2	1	3 (17.6)	0.40	0.01–9.36	0.58
Type of recruitment						
Military	2	8	10 (58.8)	1 (Reference)	-	
Civilians	6	1	7 (41.2)	0.05	0.01–0.79	0.02
Years of employment						
<1	2	5	7 (41.2)	-	-	
1–2	4	2	6 (35.3)	-	-	
2 years or more	2	2	4 (23.5)	-	-	0.41
Marital status						
Non married	3	6	9 (52.9)	1 (Reference)	-	
Married	5	3	8 (47.1)	0.32	0.03–3.11	0.35
Education level						
Bachelor	5	7	12 (70.6)	-	-	
Master	1	2	3 (17.6)	-	-	
PhD	2	0	2 (11.8)	-	-	0.46
Smoking						
No	7	9	16 (94.1)	1 (Reference)	-	
Yes	1	0	1 (5.9)	0.00	0.00–34.67	0.47
Drinking						
No	1	4	5 (29.4)	1 (Reference)	-	
Yes	7	5	12 (70.6)	0.20	0.01–2.84	0.29
Number of calls						
Less than 4 times per day	4	6	10 (58.8)	-	-	
4–5 times per day	2	2	4 (23.5)	-	-	
More than 5 times per day	2	1	3 (17.6)	-	-	0.81
Number of field deployments						
Once a week or less	3	2	5 (29.4)	1 (Reference)	-	
2–3 times a week	5	7	12 (70.6)	2.01	0.16–32.90	0.62

^†^ CI: confidence interval.

**Table 2:** Association between occupational stress, job satisfaction, and turnover intention.

Variables	Turnover Intention		Odds Ratio	95% CI ^†^	*p*-Value	Total *n* (%)
	No (*n* = 8)	Yes (*n* = 9)				
**Occupational stress**						
Role Overload	3	0	0	0–2.21	0.20	3 (17.7)
Role Insufficiency	3	5	2.00	0.21–22.15	0.64	8 (47.1)
Role Ambiguity	6	5	0.44	0.03–4.73	0.62	11 (64.7)
Role Boundary	5	1	0.09	0.01–1.26	0.049	6 (35.3)
Responsibility	1	2	1.92	0.08–123.45	1.00	3 (17.7)
Physical Environment	2	0	0	0–4.60	0.21	2 (11.8)
Job Satisfaction						
Very Unsatisfied	1	2	-	-		3 (17.7)
Unsatisfied	1	0	-	-		1 (5.9)
Partly Satisfied	6	6	-	-		12 (70.6)
Satisfied	0	1	-	-		1 (5.9)
Very satisfied	0	0	-	-	1.00	0

^†^ CI: confidence interval.

**Table 3:** Ordinal logistic regression of variables associated with turnover intention.

Variables	Estimated Coefficient	Standard Error	Adjusted Odds Ratio	95% CI ^†^	*p*-Value
Intercept	2.38	0.28	-	-	<0.01
Type of recruitment	−0.53	0.20	0.59	0.39–0.88	0.02
Occupational stress (Role boundary)	−0.35	0.21	0.70	0.47–1.06	0.11

^†^ CI: confidence interval.

## Appendix A

**Table A1:** Interpretation of the occupational role questionnaire [16,17].

Subdomain	Interpretation
Role Overload	A high score demonstrates their workload increasing and not having appropriate support.
Role Insufficiency	A high score suggests their skills are unsuitable for their work.
Role Ambiguity	A high score indicates a vague feeling of what they are expected in their work.
Role Boundary	A high score suggests conflicting sense arising from the demands of their supervisor.
Responsibility	A high score indicates a high level of responsibility for their job.
Physical Environment	A high score suggests it is highly likely to have a high level of noise or an unpleasant situation.
